# The concept of neurogenic organ dysfunction is not new and should encompass all aspects of the underlying pathophysiological mechanisms^☆^

**DOI:** 10.1016/j.mmr.2026.100052

**Published:** 2026-06-27

**Authors:** Josef Finsterer

**Affiliations:** Neurology & Neurophysiology Center, Vienna 1180, Austria

**Keywords:** Traumatic brain injury (TBI), Neurogenic organ dysfunction syndrome (NODS), Glymphatic system (GS), Immune system, Blood-brain barrier

We read with interest the article by Zhang *et al*. [1] describing neurogenic organ dysfunction syndrome (NODS) following acute traumatic brain injury (TBI). NODS is characterized by systemic complications of TBI, such as induction of the coagulation cascade, systemic inflammation, and dysfunction of the cardiovascular, respiratory, and digestive systems [1]. It leads to systemic instability caused by internal and external disturbances of the neural center [1]. The article is forward-looking, but has some limitations that should be discussed.

First of all, NODS is not a new concept for explaining the impairment of extracerebral organs by TBI, but simply a new name for an already known concept. The brain network theory was developed over 10 years ago and assumes that cerebral functions operate via interconnected, hierarchically structured networks. In this theory, networks are defined as intrinsically directed and weighted systems with heterogeneous nodes and edges [2]. Nodes represent brain regions with different microstructural properties, and edges denote connections that can act excitatorily or inhibitorily [2]. These networks evolve and can be represented in neuroscience research as static, undirected binary graphs [2]. It is also well established that cerebral networks control many fundamental systems via autonomic reflexes mediated by neural pathways in the hypothalamus, brainstem, and spinal cord, and generally regulate organ and system performance very effectively [3].

The second point is that the NODS concept does not consider important aspects of the pathophysiology of TBI. One of the mediators of organ dysfunction in acute TBI not included in the NODS proposal is the glymphatic system (GS), which plays a crucial role in the brain’s response to TBI. It is involved in the removal of necrotic, ischemic, and apoptotic tissue, as well as in the elimination of other neurotoxic metabolites and inflammatory mediators. At the same time, the function of the GS is severely impaired by TBI, transforming it from a protective mechanism into a factor in secondary brain injury. Impaired glymphatic metabolite clearance in acute TBI leads to the accumulation of proteins such as alpha-synuclein, amyloid-beta, tau, apolipoprotein E, C9orf72, superoxide dismutase, transactive response DNA binding protein 43 kDa (TDP-43), leucine-rich repeat kinase 2, huntingtin, and prion protein [4]. These proteins are associated with subsequent neurodegeneration and chronic traumatic encephalopathy. Since an acute TBI can impair not only the breakdown of metabolic products in the brain, but also nutrient supply, the distribution of substances, and the regulation of interstitial fluid, it has significant effects on sleep and the immune system. Impaired function of the GS can also cause neurodegeneration, cerebral edema, neuroinflammation, increased intracranial pressure, and secondary dysfunctions of other organs can ensue.

Thirdly, the proposed NODS concept also does not include the effects of acute TBI on the systemic and cerebral immune system. Acute TBI causes immunosuppression, leading to a reduction in white blood cells and impaired immune cell function, secondarily increasing the risk of systemic infections [5]. Acute TBI also induces chronic neuroinflammation, as the initial protective activation can persist for months or years, thus contributing to long-term cognitive impairment and neurodegenerative diseases. Acute TBI can also be responsible for dysfunction of microglia, brain-specific immune cells with a waste disposal (“recycling”) function. Suppression of microglia function by acute TBI can lead to an accumulation of cellular debris, which in turn hinders recovery. As a consequence, persistent immune dysfunction may ensue, including a reduction in T-lymphocytes and impaired cytokine production, making the immune system appear older and less efficient. Immune cells from the peripheral blood may infiltrate the brain, contributing to tissue damage and swelling rather than repairing the damage. Since acute TBI can increase cortisol levels, the immune system’s ability to destroy pathogens may be impaired.

Fourthly, the NODS proposal does not consider the effect of TBI on the blood-brain barrier. TBI can damage the blood-brain barrier [6] and consequently immune cells enter the brain and interact with neurons. This can result in the production of autoantibodies that attack neurons and glial cells, thus exacerbating secondary damage. The triggering of autoantibody production may result in cross-reactions with extra-neuronal tissue.

Until the pathophysiological basis and clinical manifestations of NODS are fully understood, numerous systemic reactions to acute TBI remain undetected and unexplained, hindering treatment. It is undeniable that TBI damages not only the brain but the entire body. However, to effectively counteract these effects, we should better explain the underlying pathophysiological concepts.

## Abbreviations

GS: Glymphatic system
NODS: Neurogenic organ dysfunction syndrome
TBI: raumatic brain injury

## Ethics approval and consent to participate

Not applicable.

## Author contributions

JF drafted the first version of the manuscript text, revised the drafts, and further edited and finalized the manuscript text. The author read and approved the final manuscript.

## Funding

Not applicable.

## Competing interests

The author declares that there is no competing interest.

## Data Availability

All data are available from the corresponding author.
